# Wireless Flexible Potentiometric Microsensors for Temperature-Compensated Sweat Electrolyte Monitoring

**DOI:** 10.1021/acsami.5c03558

**Published:** 2025-05-10

**Authors:** Jimin Lee, Leel Mazal Liberty, Ira Soltis, Kangkyu Kwon, David Chong, Youngjin Kwon, Woon-Hong Yeo

**Affiliations:** † George W. Woodruff School of Mechanical Engineering, 115724 Georgia Institute of Technology, Atlanta, Georgia 30332, United States; ‡ Wearable Intelligent Systems and Healthcare Center (WISH Center) at the Institute for Matter and Systems, 1372 Georgia Institute of Technology, Atlanta, Georgia 30332, United States; § School of Chemical and Biomolecular Engineering, 1372 Georgia Institute of Technology, Atlanta, Georgia 30332, United States; ∥ Department of Mechanical Engineering, Massachusetts Institute of Technology, Cambridge, Massachusetts 02139, United States; ⊥ School of Materials Science and Engineering, Georgia Institute of Technology, Atlanta, Georgia 30332, United States; # Wallace H. Coulter Department of Biomedical Engineering, Georgia Institute of Technology and Emory University School of Medicine, Atlanta, Georgia 30332, United States; ∇ Parker H. Petit Institute for Bioengineering and Biosciences, Georgia Institute of Technology, Atlanta, Georgia 30332, United States; ○ Korea KIAT-Georgia Tech Semiconductor Electronics Center (K-GTSEC), Georgia Institute of Technology, Atlanta, Georgia 30332, United States

**Keywords:** wearable potentiometric sensors, sweat electrolyte monitoring, temperature correction, ion-to-charge transducer, ion-selective membrane, electrochemical sensors

## Abstract

Sweat electrolyte analysis using potentiometric systems is a promising approach for continuous health monitoring. However, despite its potential, temperature-induced measurement errors remain a critical challenge, and, to our knowledge, no study has effectively addressed this issue for accurate potentiometric sensing during physiological activities. Here, we present a temperature-compensated flexible microsensor integrated with a wireless potentiometric measurement circuit for real-time sweat analysis. The wearable system features an array of microsensors for simultaneous detection of pH, Na^+^, K^+^, and skin temperature, enabling real-time dynamic temperature compensation. A PEDOT:PSS/graphene ion-to-charge transducer enhances sensitivity through superior electron acceptor properties and an expanded electroactive surface area. The incorporation of a Nafion top layer ensures 2-week-long stability by facilitating selective cation transport while mitigating sensor degradation. With temperature compensation, the wireless wearable device measures an accurate level of electrolytes under extreme temperature variations (8 to 56 °C), including outdoor exercises and exposure to dry saunas, to assess the necessity of temperature correction. This work collectively establishes a robust, high-performance platform for continuous monitoring of sweat biomarkers, thus advancing wearable diagnostic technology for personalized healthcare applications.

## Graphical abstract

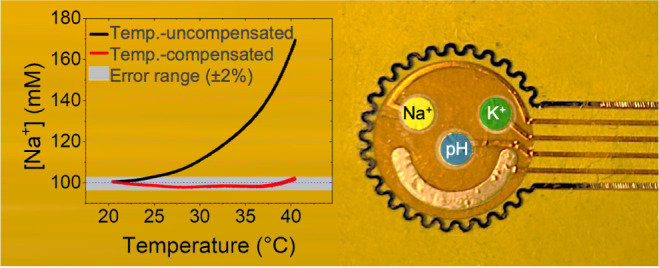

## 1 Introduction

The integration of artificial intelligence technologies into healthcare has accelerated the development of real-time, continuous biomarker monitoring and diagnostic systems from human biofluids.^1–4^ Moving beyond traditional methods such as self-administered blood tests or single-use diagnostic kits, as well as the on-site clinical visits typically required for biomarker assessment, wearable devices now enable the rapid collection and monitoring of multiday data streams seamlessly in everyday life. Technologies like continuous glucose monitoring systems illustrate the potential of wearable devices to transcend temporal and spatial limitations.^5^ To achieve continuous monitoring, advanced wearable technologies have offered the detection of pH, electrolytes, glucose, lactate, hormones, and more in biofluids.^6–14^ Among these, noninvasive health monitoring has gained significant attention for interstitial fluid sampling or sweat monitoring systems. Potentiometric sensors stand out for their ability to measure the open circuit potential between a reference electrode and a working electrode.

Despite their simplicity, the existing potentiometric sensors often overlook a critical factor: temperature. While experimental calibration curves derived from a measurement with stock solutions at room temperature are directly used to interpret on-skin monitoring results, this approach ignores the potential changes induced by actual application temperatures. This oversight introduces significant errors, especially given that Nernstian responses are inherently temperature-dependent.^15,16^ For example, even commercial pH buffer solutions provide temperature correction values to maintain accuracy, as demonstrated by a pH 10 buffer solution showing a variation from 10.19 to 9.79 across a temperature range of 5–50 °C.^17^ This 0.4 pH error highlights the critical importance of temperature compensation in healthcare monitoring. Temperature-induced errors can be particularly pronounced in scenarios involving on-skin applications, where activities such as exercise inevitably elevate skin temperature. Without real-time temperature monitoring and compensation, potential fluctuations can introduce significant errors in biomarker concentration calculations, leading to substantial mathematical inaccuracies.^18^ For instance, applying calibration curves derived from room-temperature experiments to on-body monitoring could result in errors associated with up to a 10 °C temperature differential. A recent study addressed this issue by integrating printed thermistors into ion sensor arrays for postprocessing temperature correction.^16^ However, their approach lacked dynamic skin temperature sensing during activities and did not evaluate long-term signal stability or on-body application.

Here, this study addresses these challenges by introducing a temperature-compensated flexible microsensor system designed for on-body sweat electrolyte monitoring. Unlike previous studies, this work explores the interplay between temperature and the primary sweat biomarkers, pH, Na^+^, and K^+^. The proposed system integrates a skin temperature sensor to capture real-time changes in skin temperature during various activities. Experiments ranged from moderate outdoor exercise in sub-10 °C conditions to extreme heat exposure exceeding 50 °C in a dry sauna. By tailoring calibration curves to account for temperature variations to exclude temperature effect, the sensor array achieved significantly improved accuracy. Aside from that, to enhance sensitivity and stability, the working electrode incorporates a modified ion-to-charge transducer membrane made of PEDOT:PSS/graphene and is covered with a Nafion layer. This configuration minimizes drift to below 0.1 mV over 14 consecutive days, marking a breakthrough in long-term reliability. This work sets a new standard for potentiometric multivariate sensors, offering a robust and highly accurate platform for continuous, noninvasive, on-body biomarker monitoring in wearable healthcare applications.

## 2 Results and Discussion

### 2.1 Overview of the Sweat Electrolyte Sensor Array Design

In this work, we developed a flexible microsensor system, designed for continuous sweat electrolyte monitoring using an all-in-one smiley sensor integrated with a wireless measurement circuit (Figure 1A). This system features a microsensor array capable of tracking skin temperature, pH, Na^+^, and K^+^ ion concentrations in sweat in real time. To ensure mechanical robustness and stable sensor performance during motion, the sensor adopts a multilayered structure, as illustrated in Figure 1B. The exploded schematic highlights key components, including the laser-induced graphene (LIG)-based temperature sensor, reference electrode (RE), ion-selective membranes (ISMs), and a flexible PI cover layer. Figure 1C presents representative data from continuous monitoring, demonstrating the sensor’s ability to detect real-time fluctuations in sweat electrolyte levels and skin temperature. The device’s ultrathin and flexible nature is showcased in Figure 1D, emphasizing its suitability for wearable applications that require conformal skin attachment and high mechanical stability.

**Figure 1:**
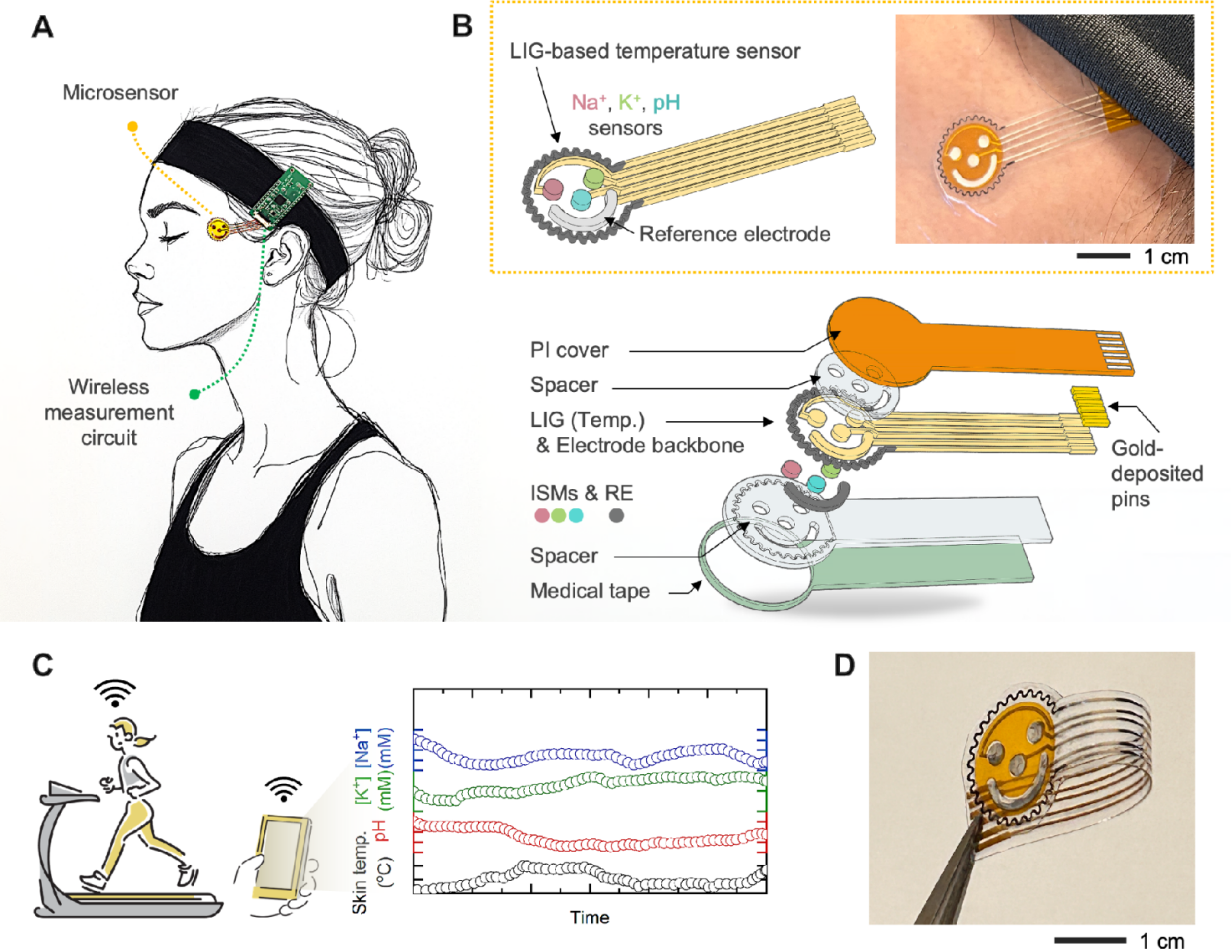
Overview of a flexible wireless microsensor system for continuous sweat electrolyte monitoring. (A) Illustration showing microsensors and a wireless circuit that are mounted on the face for continuous sweat monitoring. (B) Details of the multilayered structure of the microsensor for detecting temperature, pH, sodium (Na^+^), and potassium (K^+^). (C) Representative data measured from a subject showing wireless continuous monitoring of skin temperature, pH, Na^+^, and K^+^ levels. (D) Photo of a fabricated smiley sensor platform highlighting its ultrathin and flexible design.

### 2.2 Fabrication and Characterization of the Electrolyte Sensors

The potentiometric sensors for Na^+^, K^+^, and pH were fabricated and characterized (Figure 2). The shared Ag/AgCl reference electrode exhibited a homogeneous surface following Ag plating and subsequent chloridation, as confirmed by FE-SEM micrograph (Figure S1), ensuring stable and reproducible measurements. Additionally, the LIG-based temperature sensor demonstrated a linear response extending beyond the physiological skin temperature range, validating its accuracy in detecting real-time thermal fluctuations (Figure S2).^19,20^

**Figure 2:**
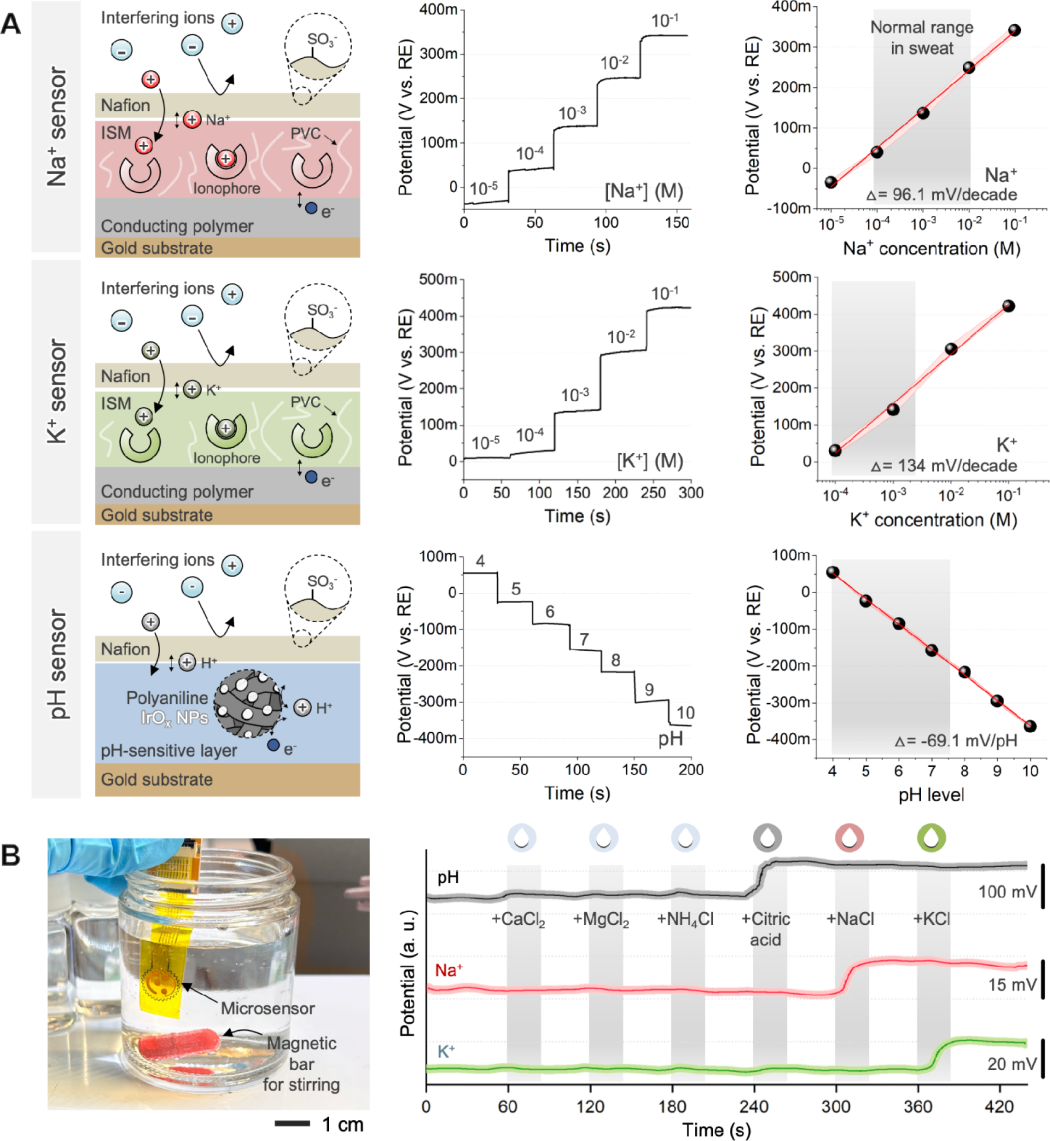
Characterization of potentiometric sensors. (A) Schematic illustration (left) of the working principle of three ion-selective electrodes for Na^+^, K^+^, and pH sensing. Corresponding potential responses (right) are plotted as a function of ion concentration, demonstrating sensor performance across physiologically relevant sweat electrolyte ranges. Error bars represent intraelectrode variability (*n* = 3). (B) Photo (left) of the microsensors immersed in a solution and measured responses (right) in the presence of a high concentration of interfering ions.

The working principle of the potentiometric sensors is illustrated in the left column of Figure 2A. Each sensor employs an ion-selective membrane (ISM) that selectively captures target ions from sweat while excluding interfering species. The Nafion finish layer, rich in sulfonate (−SO_3_^–^) functional groups, facilitates rapid cation transport, enhancing response time and preventing uncontrolled ion exchange. Additionally, an ion-to-charge transducer membrane was incorporated at the bare gold (Au)-ISM interface, amplifying potential variations and significantly improving sensor sensitivity.

The middle column of Figure 2A depicts sensor responses across different ion concentrations, demonstrating an excellent linear relationship within the physiological sweat range (Na^+^ 10^–4^ to 10^–2^ M; K^+^ 10^–4^ to 5 × 10^–3^ M). Notably, the sensitivity slopes of the Na^+^ (∼96.1 mV/dec) and K^+^ (∼134.0 mV/dec) sensors were significantly higher than the Nernstian theoretical value (∼58 mV/dec). This enhancement is attributed to the ion-to-charge transducer membrane, which improves charge transfer efficiency. For the pH sensor, a binary-phase electrode structure was employed, consisting of an electrodeposited polyaniline (PANI) layer (pernigraniline salt form) coated with iridium oxide (IrO_*x*_) nanoparticles. This composite configuration was designed to synergistically combine the advantages of each material—PANI provides mechanical robustness and stable adhesion to the electrode surface, while IrO_*x*_ contributes high pH sensitivity. Traditional single IrO_*x*_ layer often suffers from delamination and mechanical instability during prolonged use, especially under hydrated or flexible conditions. By introducing a stable PANI foundation, the binary-phase structure ensures strong adhesion of IrO_*x*_ nanoparticles and suppresses physical deterioration, enabling reliable, long-term pH sensing.^6^ The electropolymerization process of aniline is shown in Figure S3. This design enabled the sensor to maintain a constant slope (i.e., −69.1 mV/pH) across the entire pH 4–10 range, ensuring reliable measurements. Despite the absence of an additional ion-to-charge transducer layer, the super-Nernstian effect of the binary-phase composition contributed to the enhanced sensitivity.^6^ To evaluate sensor selectivity, measurements were performed in artificial sweat with the sequential addition of representative interfering ions (Figure 2B). The sensor array was immersed in artificial sweat and 0.5-M solutions of CaCl_2_, MgCl_2_, NH_4_Cl, citric acid, NaCl, and KCl were individually spiked into the solution under vigorous stirring. Despite the presence of these potential interferents commonly found in sweat, our sensor shows minimal signal variation and maintains high specificity toward respective target ions, confirming robust selectivity in complex ionic environments.

### 2.3 Study on Effective Ion-to-Charge Transducer Membrane

To optimize the sensitivity and stability of the potentiometric sensor, various ion-to-charge transducer materials were systematically evaluated, including bare Au, PEDOT:PSS, polyaniline (PANI; pernigraniline salt form), ferrocenemethanol, and PEDOT:PSS/graphene (Figures S4–S6). The effectiveness of these materials was assessed based on sheet resistance, charge transfer efficiency, and potentiometric response. Figure S4 presents the morphological characteristics and sheet resistance of different transducer materials. While bare Au exhibited high conductivity, it lacked sufficient charge transducing capability. The introduction of conducting polymers (PEDOT:PSS, PANI) and redox-active materials (ferrocenemethanol, PEDOT:PSS/graphene) significantly enhanced charge storage and ion exchange efficiency. Among these, PEDOT:PSS/graphene demonstrated the lowest sheet resistance, confirming its superior electrical conductivity and electroactive surface area. The potentiometric response of Na^+^ and K^+^ sensors utilizing different ion-to-charge transducer layers was evaluated. Figure S5A shows the dynamic potential response to stepwise Na^+^ concentration changes, while Figure S5B presents the corresponding calibration curves. Similarly, Figure S6A illustrates the response for K^+^ sensors, with the respective calibration curves shown in Figure S6B. The results reveal that PEDOT:PSS/graphene exhibited the highest sensitivity (96.1 mV/dec for Na^+^; 134.0 mV/dec for K^+^), surpassing other materials. This enhancement is attributed to its high redox capacitance, increased electroactive surface area, and superior charge transfer efficiency. Additionally, while PANI-based transducers demonstrated good stability, they exhibited higher signal drift and lower sensitivity, indicating limited long-term reliability. Ferrocenemethanol, despite showing relatively high sensitivity, exhibited irreversible potential changes (unrecovered signal drift), making it less suitable for real-time, high-resolution electrolyte monitoring. These findings confirm that PEDOT:PSS/graphene, which integrates both redox capacitance and double-layer capacitance, is the most effective ion-to-charge transducer material, providing enhanced sensor response, improved charge transfer dynamics, and minimized signal drift.^21^ These advantages and high sensor flexibility are ideal for wearable sweat electrolyte monitoring (Figure S7).

### 2.4 Dynamic Temperature Compensation via Tailored Calibration Curves

The accuracy of potentiometric ion sensors is highly susceptible to temperature variations, particularly in on-body applications where skin temperature fluctuates due to physical activity and environmental exposure. Although the Nernst equation theoretically predicts an insignificant temperature dependence (<0.1 mV/°C), empirical data show that the sensor’s material composition, geometry, and other unaccounted factors introduce substantial temperature-induced deviations, necessitating real-time correction for reliable electrolyte analysis.^16^ We analyzed physiological skin temperature variations under different activity levels to establish a compensation range. Figure 3A illustrates forehead temperature fluctuations during various activities, including walking, jogging, cardio exercises, and stretching. Additionally, to facilitate forearm sweat electrolyte monitoring, forearm skin temperature variations were also recorded (see temperature measurement points in Figure S8). The recorded skin temperature gradually increased from 30.5 to 34.3 °C, reflecting the body’s thermoregulatory response, which can significantly impact potentiometric sensor readings. Studies using infrared thermography during graded treadmill and cycling exercises have demonstrated that skin temperature typically ranges between 28 and 36 °C, and exhibits both thermal (e.g., metabolic heat, vasodilation) and nonthermal (e.g., vasoconstriction due to sympathetic activation) influences. This dynamic and region-specific nature of skin thermoregulation can significantly affect the output of on-body potentiometric sensors.^22–25^ Based on our experimental results and literature evidence, we defined a physiological skin temperature range of 20 to 40 °C as the working window for temperature-compensated calibration. To quantify the influence of temperature on sensor performance, we immersed the Na^+^, K^+^, and pH sensors in well-defined calibration solutions while gradually heating the electrolyte solution. Real-time temperature measurements of the solutions were achieved by the integrated LIG temperature sensor. The left column in Figure 3B presents the raw potential variations in response to temperature changes across different ion concentrations, showing a non-negligible drift. The top middle column highlights the magnitude of this drift, with Na^+^ sensors in 100 mM Na^+^ solution displaying a potential shift of <30 mV over a 20 °C temperature change. Using the calibration equation from Figure 2A, this drift corresponds to an apparent Na^+^ concentration increase from 100 mM to 170 mM, resulting in an overestimation of more than 60%. Similarly, K^+^ sensors exhibited +30% variation, and pH measurements showed significant fluctuations, further emphasizing the necessity of temperature correction in potentiometric sensing. To mitigate these errors, we developed a temperature compensation algorithm using tailored calibration curves (fitting equations shown in Figures S9–S11). Empirical data demonstrated an excellent fit with the equation *V* = *a* + *bT* ^*c*^, confirming the reliability of our compensation model in accurately adjusting sensor readings across varying temperatures. The right column in Figure 3B demonstrates the effectiveness of this approach, where the compensated data (red) remain within an error margin of ±2%, ensuring accurate electrolyte concentration measurements despite temperature fluctuations. These results highlight the critical role of temperature correction in wearable potentiometric sensors, significantly improving their reliability for real-time, on-body sweat analysis.

**Figure 3:**
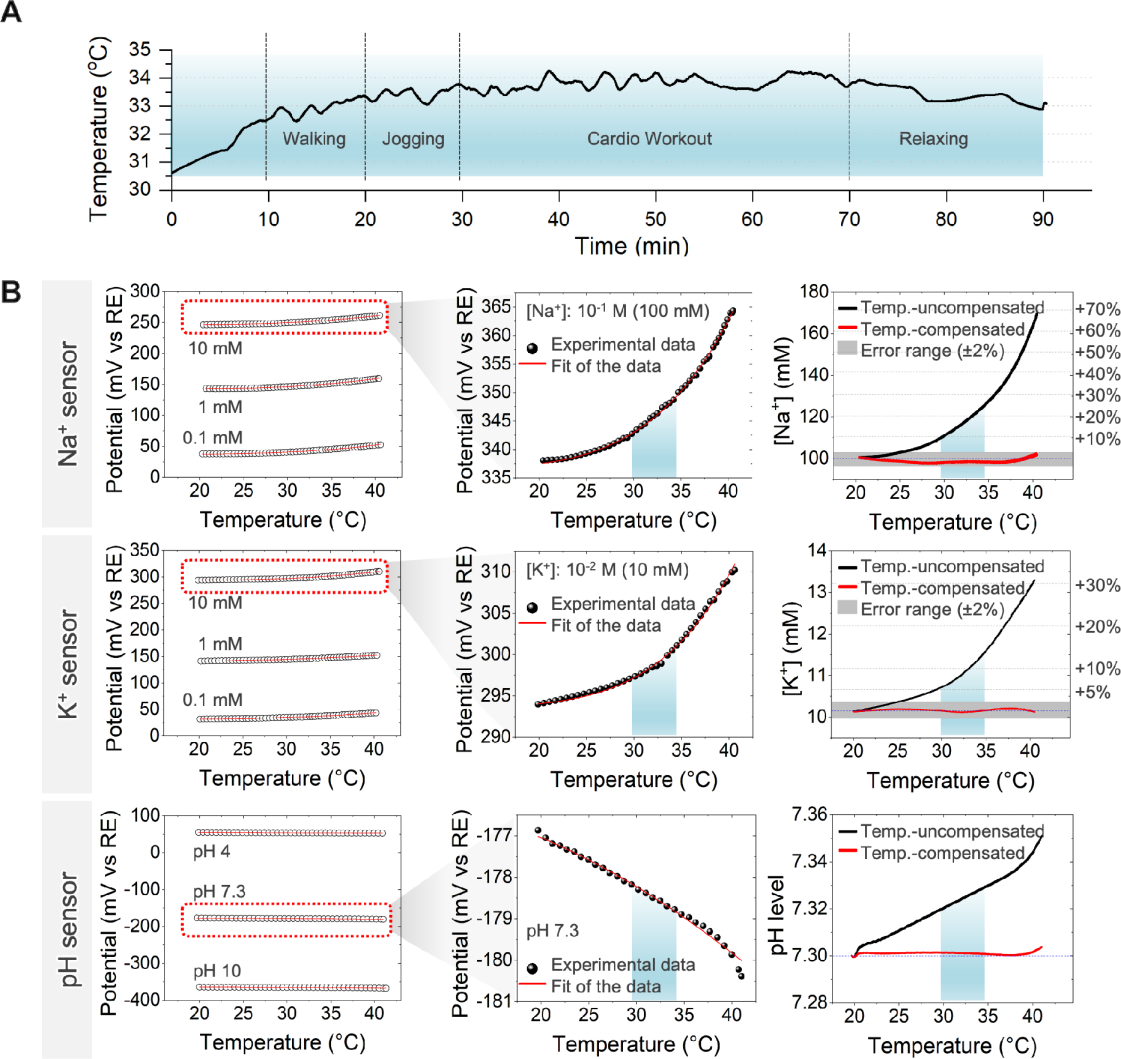
Temperature-compensated sensor calibration. (A) Measurement of skin temperature variations during different activities, to define physiological temperature changes for sensor calibration. (B) Characterization of Na^+^, K^+^, and pH sensors under varying temperature conditions. The left column presents potential variations measured in well-defined stock calibration solutions with known ion concentrations. The middle column shows potential drifts derived from temperature-dependent sensor responses. The right column illustrates the effect of temperature compensation, where the temperature-corrected data (red) remain within an error range of ± 2%, demonstrating improved accuracy.

### 2.5 On-Body Sweat Monitoring

To evaluate the real-world performance of the temperature-compensated flexible microsensor, on-body tests were conducted under various conditions, including moderate exercise, rest, and extreme temperature exposure. The sensors were placed on the forehead and forearm to monitor Na^+^, K^+^, pH, and skin temperature during different activities (Figure 4A–C), ensuring continuous, noninvasive sweat analysis in dynamic environments (see the sensing system integrated into a sports headband in Figure S12). Sweat electrolyte monitoring was performed during an interval training session consisting of warm-up, cardio exercise, juice consumption, and cool-down phases. Figure 4D,E display the recorded Na^+^, K^+^, pH, and skin temperature variations for the forehead and forearm, respectively (see the multiaxis plots in Figures S13 and S14, respectively). It is important to note that, prior to the onset of perspiration, the sensors and reference electrode are not fully wetted by a continuous ionic medium, making accurate open-circuit potential (OCP) measurements infeasible. Therefore, while data recording began immediately upon sensor placement, only the stabilized sensor signals—collected after visible sweat formation and signal stabilization—were used for concentration analysis and visualization in the figures. Throughout the exercise, skin temperature increased gradually, influencing the raw potential response of the electrolyte sensors. The dynamic temperature compensation algorithm successfully corrected the signal, ensuring accurate electrolyte concentration readings. Notably, Na^+^ and K^+^ concentrations exhibited characteristic variations associated with sweat secretion and reabsorption, while pH remained stable within the physiological range. Similar sensor systems reported in the literature have demonstrated comparable electrolyte ranges but with greater variability (e.g., Na^+^: 20–100 mM; K^+^: 3–10 mM; pH: 4–7.5).^26^ To examine the sensor’s robustness in extreme environmental conditions, healthy subjects underwent a series of activities in environments with substantial temperature fluctuations. Figure 5A–C outlines the experimental setup, which included an outdoor workout at 8 °C, indoor rest at 19 °C, and dry sauna exposure at 56 °C. The results in Figure 5D illustrate the recorded Na^+^, K^+^, pH levels, and forehead skin temperature throughout the session (see the multiaxis plot in Figure S15). The raw potentiometric data exhibited significant deviations due to temperature changes, particularly during the dry sauna phase, where the skin temperature exceeded 40 °C. As the body’s thermoregulatory mechanisms struggled to compensate for the extreme heat, skin temperature continued to rise, further influencing sensor readings^27^ Figure 5E highlights the critical impact of temperature compensation, comparing the sensor outputs before (hollow dots) and after (filled dots) correction. Without compensation, the electrolyte concentration estimates showed large errors, with Na^+^ overestimated by 100% and K^+^ by 300%. After applying temperature correction, the measured values aligned within ± 2% of expected concentrations. To further validate the accuracy of the compensated sensor readings, sweat samples were collected after 20 min in the dry sauna and analyzed using ICP-MS (for Na^+^ and K^+^) and a pH colorimetric sensor (Figure 5F). The temperature-compensated measurements closely matched the ICP-MS results, while uncompensated values exhibited significant deviations, reinforcing the necessity of real-time temperature correction for wearable potentiometric sensors.

**Figure 4:**
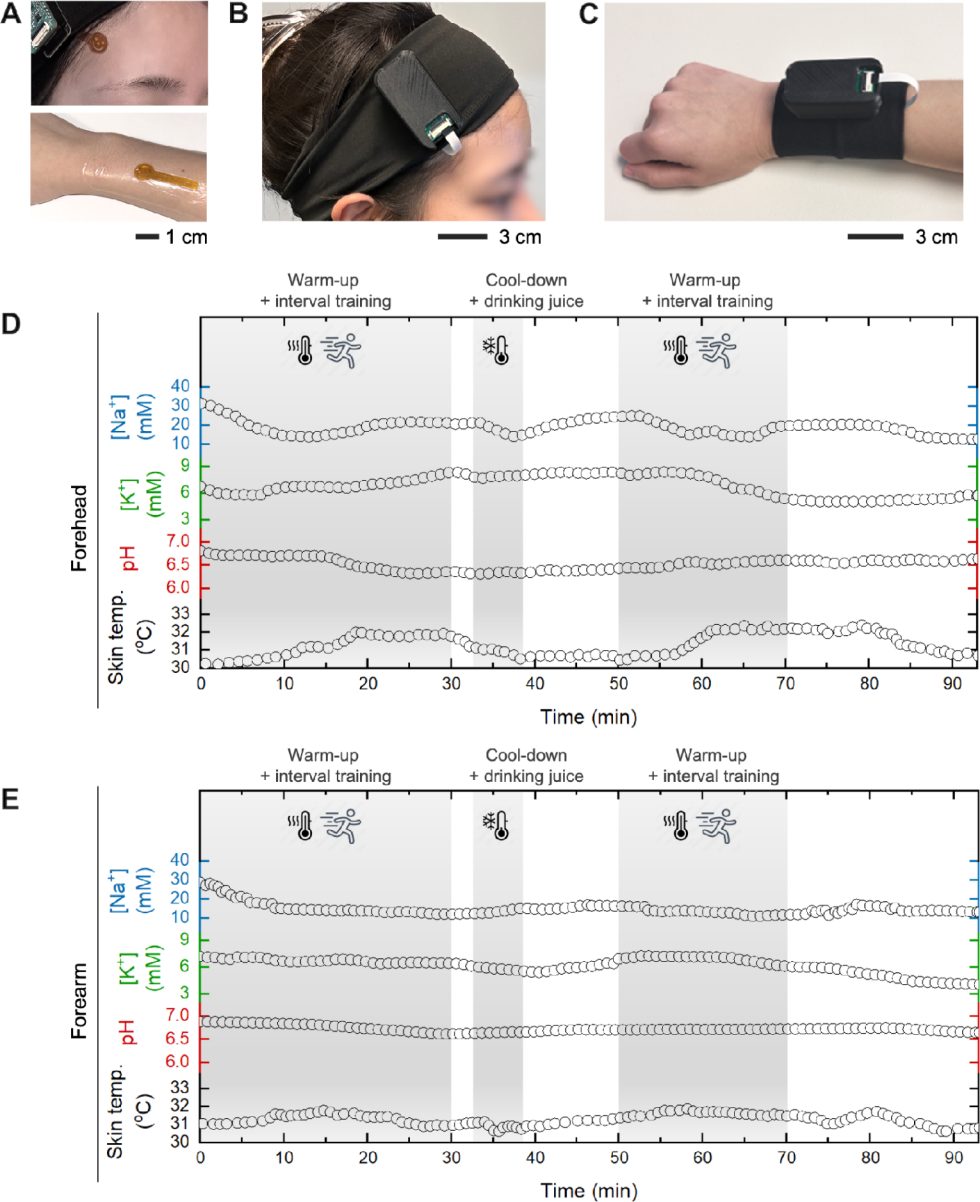
On-body monitoring of sweat electrolyte levels. (A–C) Photos of fabricated flexible microsensors (A) and wireless circuits on the forehead (B) and the forearm (C) for sweat electrolyte monitoring. (D,E) On-body measurements of skin temperature, pH, Na^+^, and K^+^ concentrations recorded during interval training sessions for the forehead device (D) and forearm device (E).

**Figure 5:**
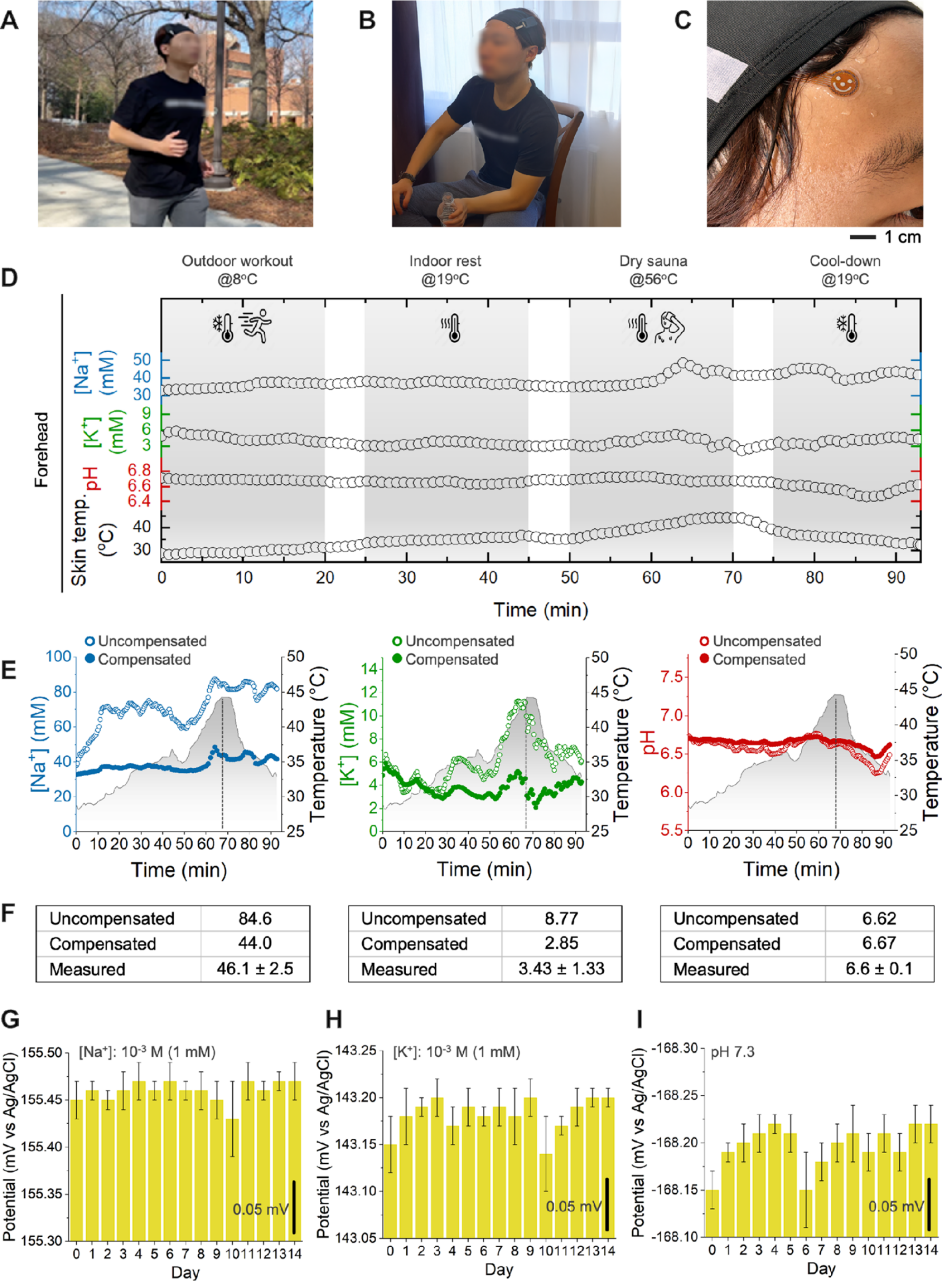
On-body monitoring of sweat electrolyte levels under harsh temperature conditions. (A–C) Experimental setup for outdoor workout at 8 °C (A), indoor rest at 19 °C (B), and dry sauna exposure at 56 °C (C) to simulate extreme temperature variations. (D) On-body monitoring results of skin temperature, pH, Na^+^, and K^+^ concentrations during the activity. (E) Comparison of potentiometric sensor data before (hollow dots) and after (filled dots) temperature compensation, demonstrating the necessity of correction for accurate electrolyte measurements. (F) Validation of temperature compensation using sweat samples collected after 20 min in the dry sauna, analyzed via ICP-MS (for Na^+^ and K^+^) and pH colorimetric sensor. The results show that temperature-compensated values align closely with the ICP-MS measurements, whereas uncompensated values exhibit significant deviations, implying the importance of temperature compensation in potentiometric sweat electrolyte sensing. (G–I) Long-term stability assessment of the sensors over 14 consecutive days in well-defined calibration solutions (1 mM Na^+^, 1 mM K^+^, and pH 7.3), showing minimal potential drift, indicating reliable and consistent sensor performance.

### 2.6 Evaluation of Long-Term Stability

The long-term stability of the sensors was assessed over 2 weeks using well-defined calibration solutions (1 mM Na^+^, 1 mM K^+^, and pH 7.3) in both PBS and artificial sweat solutions (Figure 5G–I) . The results obtained from artificial sweat solutions are in Figure S16. The sensors exhibited minimal potential drift (<0.15 mV over 2 weeks), demonstrating excellent stability and reliability compared to conventional ion-selective electrodes. Notably, the incorporation of PEDOT:PSS/graphene as an ion-to-charge transducer material, combined with a Nafion protective layer, played a crucial role in enhancing sensor longevity.^28,29^ Nafion, coated over the ISM, helped maintain a stable electrode surface, preventing fluctuations caused by environmental factors and potential ion exchange imbalances. Artificial sweat, in contrast to PBS, contains a broader range of ionic species. Despite this increased complexity, the sensors maintained a stable drift profile, suggesting robust resistance to interference from multiple ionic components. It is worth noting that the presence of various ions in artificial sweat did not compromise sensor stability, further confirming the effectiveness of the Nafion protective layer and PEDOT:PSS/graphene transducer membrane in mitigating sensor degradation. These results underscore the suitability of the proposed sensor system for long-term wearable biomarker monitoring applications, ensuring reliable performance in real-world conditions. Table 1 compares existing sweat electrolyte sensors, highlighting key factors such as temperature compensation, sensitivity, potential drift, and stability (visualized in Figure S17). Unlike most previously reported sensors, which lack temperature correction, our system integrates compensation, significantly enhancing measurement accuracy in dynamic conditions. Our sensor demonstrates high sensitivity (Na^+^: 96.1 mV/dec, K^+^: 134.0 mV/dec, pH: −69.1 mV/pH) while achieving exceptionally low potential drift (<0.15 mV over 2 weeks), outperforming conventional designs that often exhibit higher drift rates. The incorporation of graphene into the PEDOT:PSS matrix further enhances stability and charge transfer efficiency. Additionally, most conventional sensors lack a protective overlay, making them prone to ion leaching and degradation. In contrast, our Nafion protective layer prevents ion loss, improving sensor longevity and reliability. These results underscore the superior long-term performance and robustness of our temperature-compensated microsensor, making it an ideal candidate for on-body sweat monitoring in wearable healthcare applications.

**Table 1:** Performance Comparison Between Wearable Sensors for Sweat Electrolyte Monitoring

ref.	Target	Temperature compensation	Sensitivity (mV/decade for Na and K) (mV/pH for pH)	Potential drift (mV/h)	Stability in sweat	Ion-to-charge materials
This work	Na^+^, K^+^, H^+^	Yes	96.1 (Na^+^);	<0.15 mV/2 week	2 weeks	PEDOT:PSS/graphene
			134.0 (K^+^);			
			69.1 (pH)			
^30^	Na^+^, K^+^	-	98.3 (Na^+^);	-	-	-
			99 (K^+^)			
^31^	Na^+^, H^+^	-	54.480 (Na^+^);	<1 mV/40 min	-	PEDOT:PSS
			55.731 (K^+^)			
^32^	Na^+^	-	56.58 (Na^+^)	0.22 mV/h	2 h	Gold nanodendrites
^26^	Na^+^, K^+^, H^+^	-	53.5 (Na^+^);	<0.6	-	MWCNTs
			57.6 (K^+^);			
			54.5 (pH)			
^21^	Na^+^, H^+^	-	59.64 (Na^+^);	6.20 μV/h (Na^+^)		NPCs@rGO950
			60.22 (K^+^)	5.41 μV/h (K^+^)		
^33^	Na^+^, K^+^, H^+^	-	59.7 (Na^+^);	0.2 – 1 mV/h	-	-
			57.8 (K^+^);			
			54.7 (pH)			
^34^	Na^+^, K^+^	-	42.5 (Na^+^);	0.02 – 0.04 V/2 week	-	PEDOT:PSS
			51.1 (K^+^)			
^35^	Na^+^	-	59.3 (Na^+^)	–	-	-
^36^	Na^+^, H^+^	-	56 (Na^+^);		-	Carbon black
			80 (pH)			
^37^	K^+^, H^+^	-	64.169 (K^+^);	-	-	Polyaniline
			46.331 (pH)			
^38^	Na^+^	-	66.2 (Na^+^)	-	-	-
^9^	Na^+^, K^+^	-	66 (Na^+^);	0.04 mV/h	100 h	-
			59.6 (K^+^)			

## 3 Conclusion

Thia paper reports on a temperature-compensated flexible microsensor system for on-body sweat electrolyte monitoring, integrating potentiometric Na^+^, K^+^, and pH sensors with a temperature sensor on a flexible substrate. The system effectively corrects temperature-induced errors, enabling accurate, continuous monitoring. The high-resolution measurement device (1 Hz acquisition, up to 100 °C) validates its performance with buffer solutions up to 40 °C, ensuring reliability by excluding temperature-dependent drift via tailored calibration curves. Without compensation, the Na^+^ and K^+^ sensors exhibit over 100% variation, leading to significant inaccuracies. After applying temperature correction, the sensor readings closely match ICP-MS measurements, confirming the necessity of temperature adjustment. The sensors demonstrate excellent long-term stability, with a minimal drift of <0.15 mV over 2 weeks, aided by a PEDOT:PSS/graphene transducer membrane and a Nafion protective layer. Notably, stable performance is maintained even in artificial sweat, despite its complex ionic composition. These results highlight the importance of temperature correction in wearable potentiometric sensors, paving the way for accurate, continuous sweat monitoring in personalized healthcare, sports performance tracking, and hydration assessment.

## 4 Experimental Section

### 4.1 Fabrication of Flexible Microsensor Array

The flexible microsensor array was fabricated using laser cutting, followed by electroplating of palladium (Pd) for interconnects and pin connections and gold (Au) for the electrodes onto a copper foil substrate. Two patterned polyethylene terephthalate (PET) films (McMaster-Carr) were prepared to serve as the bottom and top spacers. The top layer was then aligned and dry-transferred onto the bottom layer using a heat press for assembly. Before integrating the bottom spacer, which serves as a sweat reservoir, electrodeposition and drop-casting steps were performed to fabricate the potentiometric sensors. The final assembled array was securely attached to the skin using medical tape (468MP, 3M).

### 4.2 Temperature Sensor Preparation

A polyimide (PI) film was attached to a spin-coated PDMS layer (Sylgard 184, Dow Corning) on a glass slide, followed by the fabrication of a laser-induced graphene (LIG) electrode using a pyrolytic ultraviolet laser (Alabama UV Laser, 355 nm). The LIG was then transferred onto a freestanding PDMS layer incorporating Pd/Cu interconnect lines and securely attached using silver epoxy (8331D, MG Chemicals) to ensure high electrical conductivity.

### 4.3 Shared Reference Electrode Preparation

A 5 μL of 0.1 M ferric chloride (FeCl_3_, 97%, Aldrich) solution was drop-cast onto the silver (Ag) finish on the bare gold (Au) electrode for chloridation, and then rinsed with deionized water. Next, a 5 μL of polyvinyl butyral (PVB, Aldrich) salt coating, enriched with potassium chloride (KCl, 99.0%, Aldrich) and silver nitrate (AgNO_3_, 98%, Aldrich), was drop-cast onto the Ag/AgCl surface and left to dry overnight. A 5 μL of Nafion (Nafion perfluorinated resin solution, Aldrich) layer was applied and dried at 60 °C for 30 min to prevent KCl leaching.

### 4.4 Potentiometric pH Sensor Preparation

A binary-phase pH sensor was fabricated as described elsewhere.^6^ Polyaniline (PANI) was electrodeposited onto a cleaned Au electrode using a precursor solution containing 0.25 M aniline monomer (99.5%, Aldrich) and 0.5 M H_2_SO_4_ (95.0–98.0%, Aldrich) in distilled water. The deposition was performed via cyclic voltammetry (scan rate: 50 mV/s, 20 cycles) using a three-electrode potentiostat (Interface 1010 E, Gamry Instruments). After rinsing, the electrode was directly immersed in an iridium oxide (IrO_*x*_) precursor solution comprising 4.5 mM iridium tetrachloride (IrCl_4_, 99.95%, Thermo Fisher), 130 mM hydrogen peroxide (H_2_O_2_, 30%, Merck), and 40 mM oxalic acid dihydrate (C_2_H_2_O_4_.2H_2_O, >99%, Aldrich), with the pH-adjusted to 10.5 using potassium carbonate (K_2_CO_3_, anhydrous, >99%, Aldrich). The solution was stabilized at room temperature for 2 days and then stored at 4 °C. IrO_*x*_ was electrodeposited via linear sweep voltammetry (0.0–1.3 V vs Ag/AgCl, 50 mV/s, 200 pulses). Following deposition, a diluted Nafion solution (25 wt % in alcohol) was applied as a cation-selective membrane and dried overnight. Rich sulfonate groups (−SO_3_^–^) of Nafion facilitate preferential transport of cations such as H^+^, Na^+^, and K^+^, while simultaneously reducing anion interference and enhancing the stability and biocompatibility of the sensing layer.

### 4.5 Potentiometric Na^+^ and K^+^ Sensor Preparation

The sodium ion (Na^+^) selective cocktail was prepared by dissolving 4 mg sodium ionophore X (4-*tert*-Butylcalix­[4]­arenetetraacetic acid tetraethyl ester, 97%, Aldrich), 2.20 mg sodium tetrakis­[3,5-bis­(trifluoromethyl)­phenyl]­borate (NaTFPB, Aldrich), 131.8 mg polyvinyl chloride (PVC, high molecular weight, Aldrich), and 261.5 mg bis­(2-ethylhexyl) sebacate (DOS, > 97%, Aldrich) in 3 mL tetrahydrofuran (THF, > 99.0%, Aldrich). Similarly, the potassium ion (K^+^) selective cocktail was prepared by dissolving 20 mg valinomycin (>99.0%, Aldrich), 5 mg potassium tetrakis­[3,5-bis­(trifluoromethyl)­phenyl]­borate (KTFPB, Aldrich), 300 mg PVC, and 700 mg DOS with 3 mL THF. Five μL of each selective cocktail solution was then drop-cast onto the ion-to-charge transducer membrane of the respective working electrode and left to dry overnight. After complete drying, a diluted Nafion solution was applied to each electrode as a finishing layer.

### 4.6 Ion-to-Charge Transducer Membrane Study

To evaluate various ion-to-charge transducer materials, different layers were introduced onto the bare Au electrode before drop-casting the ion-selective membrane (ISM). PANI (pernigraniline salt form) was electrodeposited onto the Au surface using a method similar to that employed in pH sensor fabrication. PEDOT:PSS (1.5% aqueous dispersion, neutral pH, Aldrich) was directly coated onto the Au surface without dilution. Ferrocenemethanol (97%, Aldrich) was dissolved in ethanol at an appropriate concentration and 5 μL of the solution drop-cast onto the Au electrode. Additionally, PEDOT:PSS was mixed with graphene flakes and dispersed using ultrasonication for several hours to prepare a PEDOT:PSS/graphene dispersion, which was subsequently drop-cast onto the bare Au electrode to form a thin-film layer. The electrodes were then baked in an oven at 120 °C for 1 h to remove residual moisture. Finally, ISM and Nafion layers were applied to complete the sensor fabrication.

### 4.7 Material Characterization

The surface microstructure of the ion-to-charge transducer membranes and the Ag/AgCl reference electrode was examined using a field-emission scanning electron microscope (FE-SEM, SU8230, Hitachi). Additional surface observations were performed using a digital microscope (VHX-7000, Keyence). The sheet resistance of each ion-to-charge transducer membrane was measured using a four-point probe system (SYS-301, Signatone) to evaluate electrical properties. To validate the accuracy of the temperature-compensated sensor readings, sweat samples were collected, diluted, and analyzed using inductively coupled plasma mass spectrometry (ICP-MS, iCAP RQ, Thermo Scientific) for Na^+^ and K^+^ and a pH colorimetric sensor (pH paper dispenser, range 5.5–8.0, Hydrion).

### 4.8 Standard Solution Test

Stock solutions of Na^+^ and K^+^ were prepared by dissolving sodium chloride (NaCl) and potassium chloride (KCl) in pure deionized water. Serial dilutions were performed as needed to achieve a concentration of 10^–5^, 10^–4^, 10^–3^, 10^–2^ and 10^–1^ M, respectively. Buffer solutions with pH values ranging from 4 to 10 (Aldrich) were used without further modification. Artificial sweat was prepared according to the reference test method EN 1811:2011 by mixing NH_4_OH solution (134 mM), urea (10 mM), NaCl (27 mM), KCl (6.1 mM), Na_2_SO_4_ (0.41 mM), choline chloride (143 mM), L­(+)-ascorbic acid (10.2 mM), D­(+)-glucose (0.17 mM), and L­(+)-lactate solution (188 mM) in distilled water. The pH was adjusted to 6.5 using 0.1 M HCl. Minor constituents, including certain vitamins, nitrogenous compounds, organic acids, carbohydrates, and some ionic components, were omitted, retaining only key chemical components. The sensor electrodes were characterized using open-circuit potential measurements performed with a three-electrode potentiostat (Interface 1010E, Gamry Instruments). The effect of temperature on sensor performance was evaluated using a ceramic hot plate (Thermo Fisher Scientific). Concentration values were interpolated from sensor-specific calibration curves using a log–linear fitting model.

### 4.9 Wireless Measurement

The wireless measurement circuit adopts a compact, low-power data acquisition system. This system comprises an AD5941 electrochemical front-end for potentiometric signal capture and an ADG804 analog multiplexer that enables sequential measurement of multiple sensor inputs. The digitized signals are transmitted via SPI to an ESP32 Feather microcontroller, which supports both Wi-Fi and Bluetooth communication protocols. For this study, Wi-Fi was utilized for real-time data transmission to either a cloud server for direct upload and processing or to a local device via USB for visualization and postanalysis. The circuit is powered by a rechargeable 3.7 V lithium-polymer battery or wall power through a USB interface, with integrated power management and charging circuitry. This modular wireless platform ensures seamless integration with microsensors for continuous, untethered sweat analysis.

### 4.10 Human Subject Study

A few healthy subjects participated in the study. The experimental protocol (IRB2025-391) was approved by the Georgia Tech Institutional Review Board, ensuring compliance with ethical research standards. In accordance with ethical guidelines, all participants provided written informed consent before the study.

## Supplementary Material



## Data Availability

The data that support the findings of this study are available from the corresponding author upon reasonable request.

## Notes

Morphology of the reference electrode (Figure S1), design and calibration of LIG-based temperature sensor (Figure S2), electro-polymerization process of polyaniline (Figure S3), material comparison of ion-to-charge transducer membranes including bare Au, PEDOT:PSS, PANI, ferrocenemethanol, and PEDOT:PSS/graphene (Figure S4), evaluation of ion-to-charge transducers for Na^+^ and K^+^ sensors (Figures S5 and S6), mechanical flexibility assessment of the sensor (Figure S7), skin temperature measurement points for on-body experiments (Figure S8), temperature-dependent potential profiles for Na^+^, K^+^, and pH sensors (Figures S9–S11), wireless circuit architecture and data acquisition system (Figure S12), real-time on-body monitoring of sweat electrolytes and skin temperature on forehead and forearm during physical activity (Figures S13 and S14), on-body sensor performance under extreme thermal conditions (Figure S15), long-term stability testing of Na^+^, K^+^, and pH sensors in PBS and artificial sweat over 14 days (Figure S16), and comparative radar plot analysis of sensor performance against existing literature (Figure S17) (PDF)

## Abbreviations

OCP: open circuit potential
RE: reference electrode
WE: working electrode
LIG: laser-induced graphene
ISM: ion-selective membrane
PANI: polyaniline
