# Supplementary material for: Wireless Flexible Potentiometric Microsensors for Temperature-Compensated Sweat Electrolyte Monitoring
Source: ACS Appl Mater Interfaces. 2025 May 10;17(25):36345–55. doi: 10.1021/acsami.5c03558 (PMC12203469; doi:10.1021/acsami.5c03558)
Supplement: Supplementary file 1 [file am5c03558_si_001.pdf]

Supporting Information

Wireless Flexible Potentiometric Microsensors for Temperature-Compensated Sweat Electrolyte Monitoring

Jimin Lee[1,2], Leel Mazal Liberty[3], Ira Soltis[1,2], Kang Kyu Kwon[2,4], David Chong[1,2], Youngjin Kwon[2,5], Woon-Hong Yeo[1,2,6,7,8*]

1 George W. Woodruff School of Mechanical Engineering, Georgia Institute of Technology, Atlanta, GA 30332, USA
2 Wearable Intelligent Systems and Healthcare Center (WISH Center) at the Institute for Matter and Systems, Georgia Institute of Technology, Atlanta, GA 30332, USA
3 School of Chemical and Biomolecular Engineering, Georgia Institute of Technology, Atlanta, GA 30332, USA
4 Department of Mechanical Engineering, Massachusetts Institute of Technology, Cambridge, MA 02139, USA
5 School of Materials Science and Engineering, Georgia Institute of Technology, Atlanta, Georgia 30332, USA
6 Wallace H. Coulter Department of Biomedical Engineering, Georgia Institute of Technology and Emory University School of Medicine, Atlanta, GA 30332, USA
7 Parker H. Petit Institute for Bioengineering and Biosciences, Georgia Institute of Technology, Atlanta, GA 30332, USA
8 Korea KIAT-Georgia Tech Semiconductor Electronics Center (K-GTSEC), Georgia Institute of Technology, Atlanta, Georgia 30332, USA
*Corresponding author: Woon-Hong Yeo, whyeo@gatech.edu

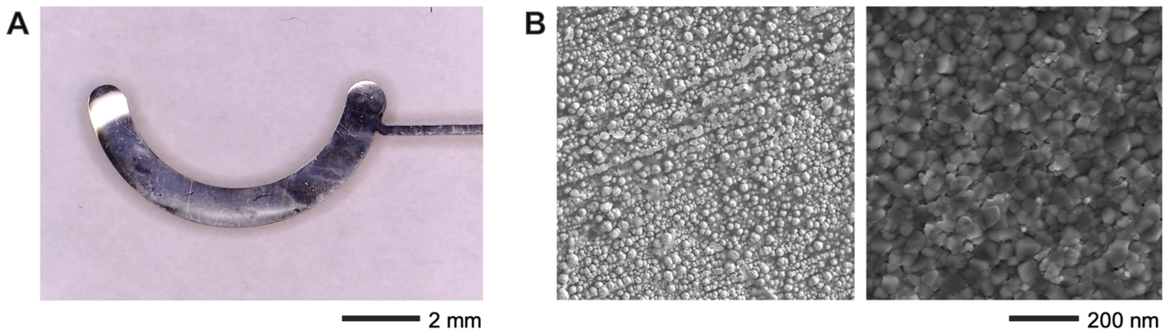

Figure S1. Morphology of the reference electrode.

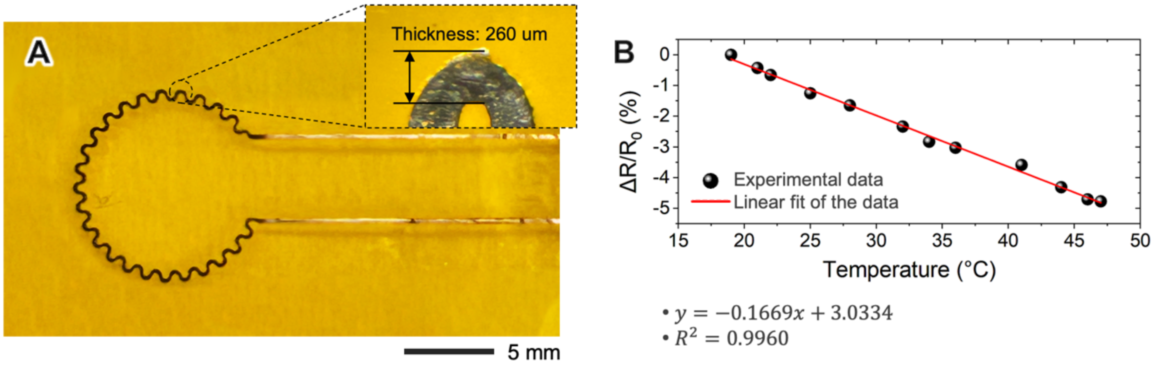

Figure S2. LIG-based temperature sensor and a corresponding calibration curve.

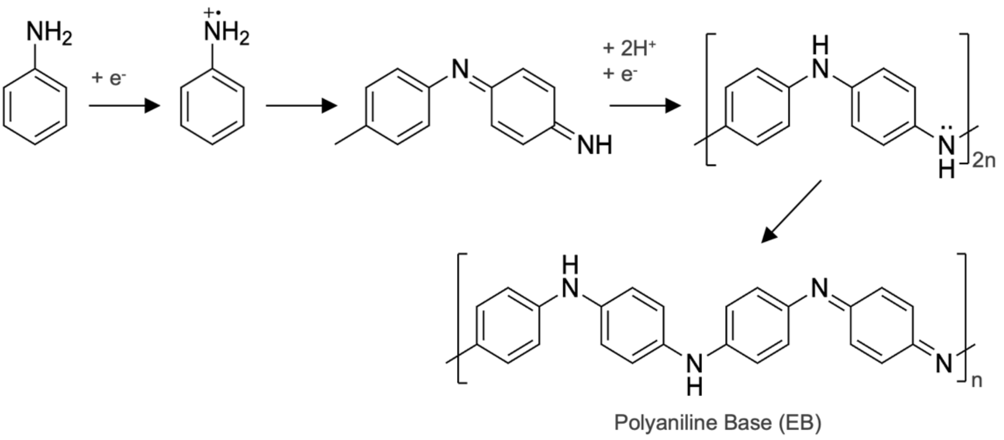

Figure S3. Electro-polymerization process of aniline.

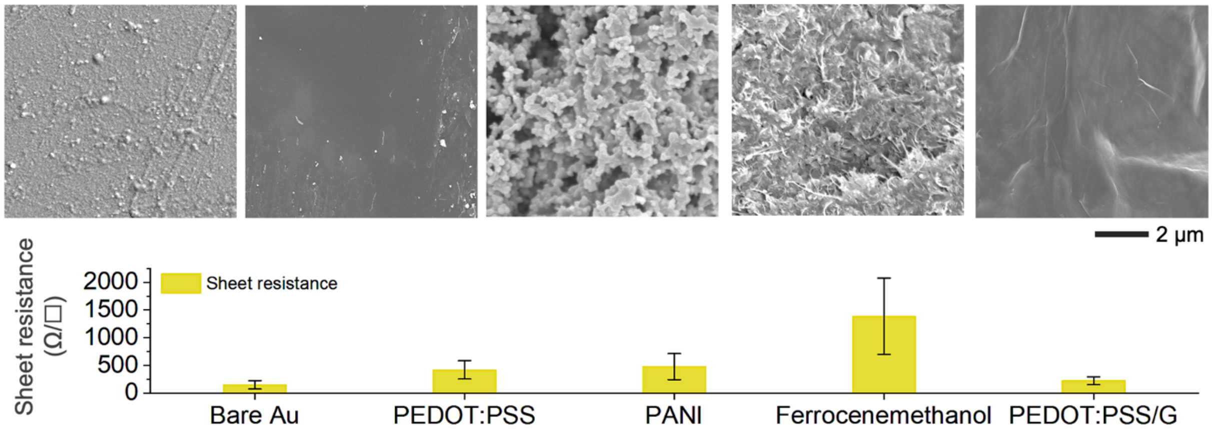

Figure S4. Characterization of Ion-to-charge transducer. Bare Au, PEDOT:PSS, polyaniline (PANI; pernigraniline salt form), ferrocenemethanol, and PEDOT:PSS/graphene were chosen a s a potential ion-to-charge transducer membrane.

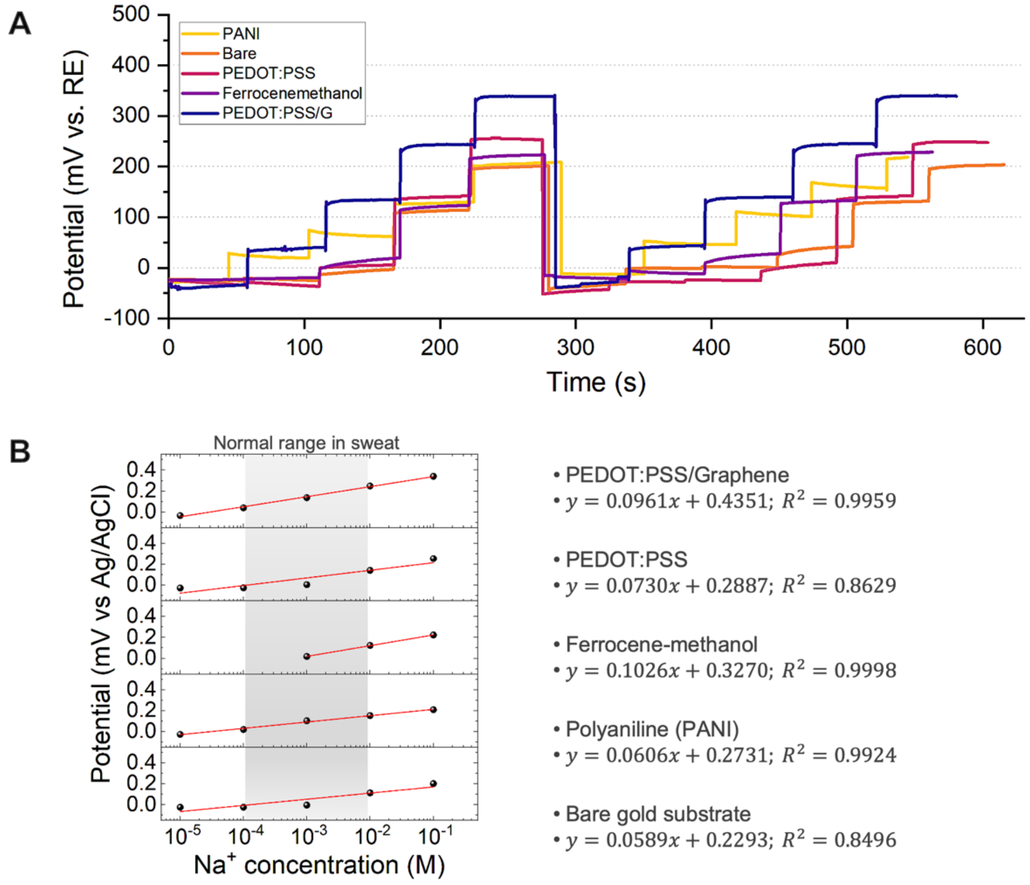

Figure S5. Evaluation result of ion-to-charge transducer membrane for Na^+ sensor.

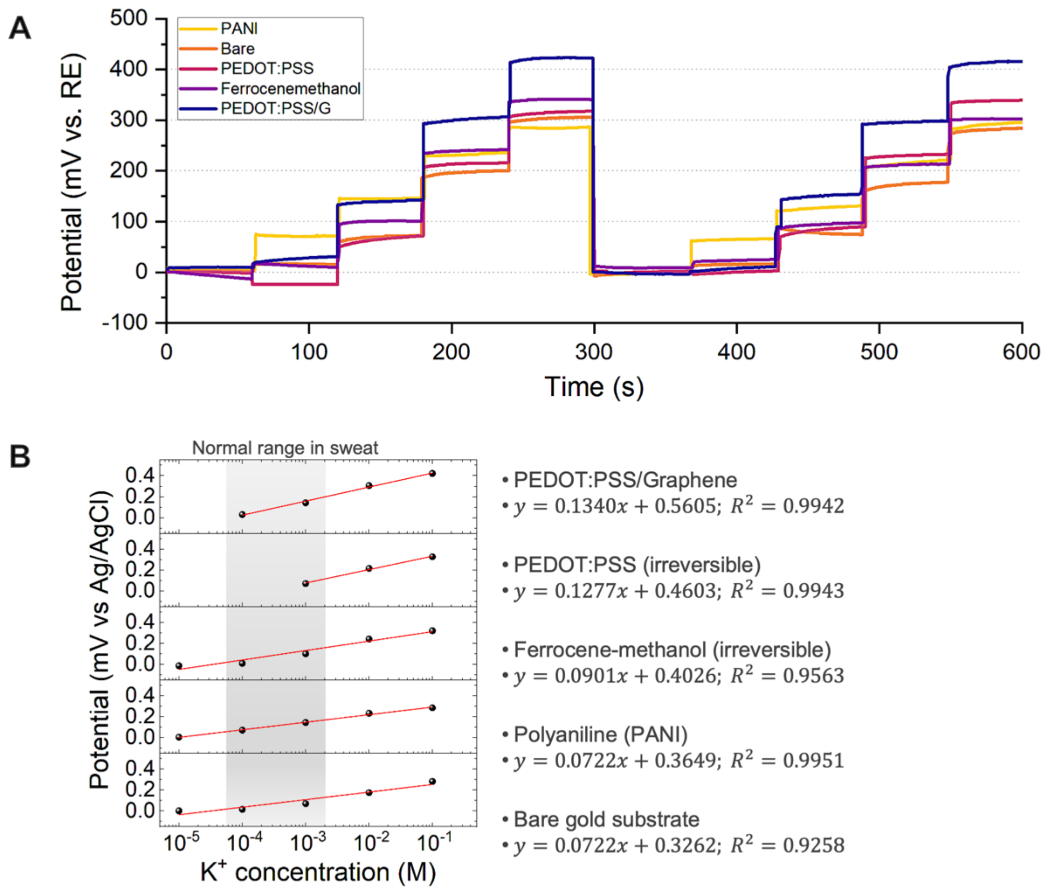

Figure S6. Evaluation result of ion-to-charge transducer membrane for K^+ sensor.

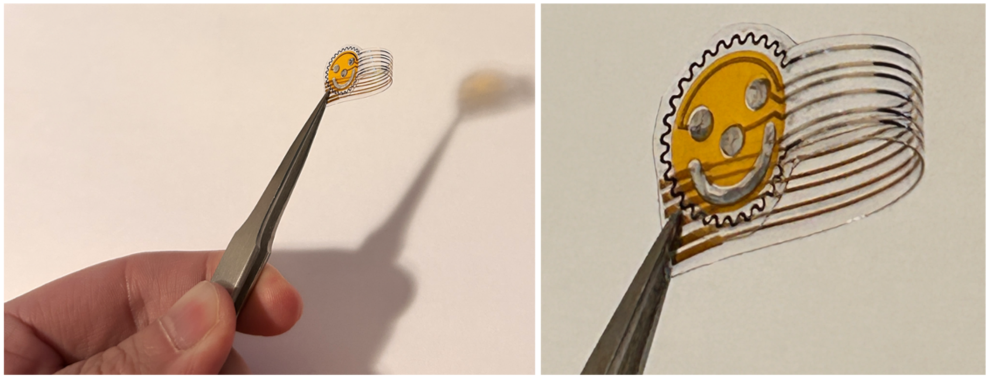

Figure S7. Flexibility of the sensor.

A B

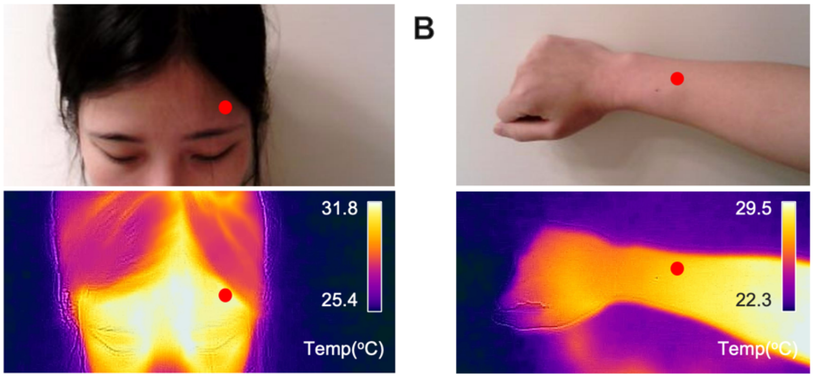

Figure S8. Temperature measurement points.

A

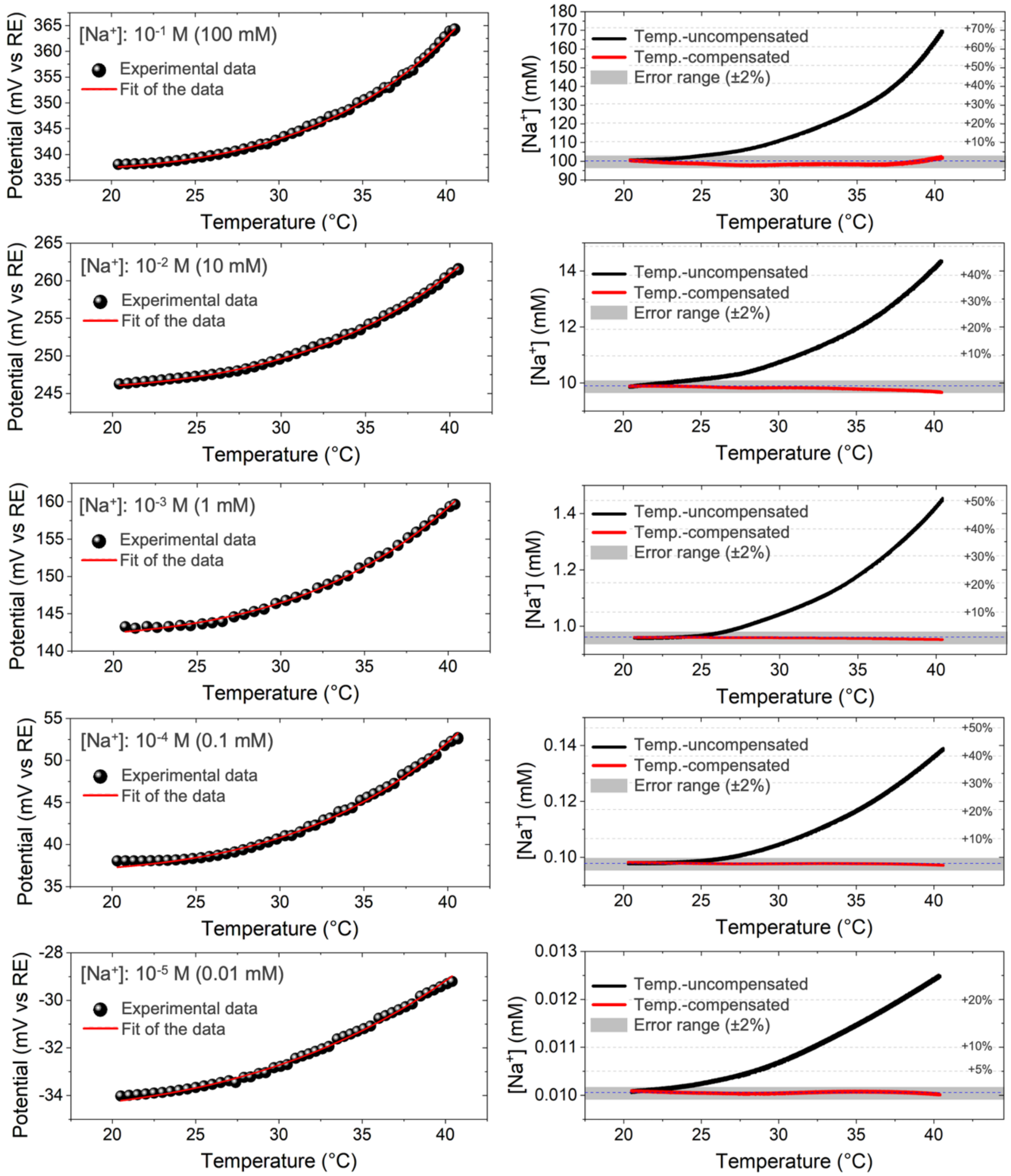

B

Equation	$y = a + bx^c$				
$[Na^+]$ (M)	10^{-5}	10^{-4}	10^{-3}	10^{-2}	10^{-1}
a	-0.72451 ± 0.01477	-1.69805 ± 0.04198	-1.83337 ± 0.0453	-1.25185 ± 0.03403	-2.67093 ± 0.05343
b	1.28107E-5 ± 1.07974E-6	1.16989E-6 ± 1.39957E-7	1.33688E-7 ± 1.72626E-8	1.38115E-6 ± 1.29968E-7	7.49699E-6 ± 6.16801E-7
c	3.51915 ± 0.02241	4.46601 ± 0.03219	5.26403 ± 0.03597	4.43529 ± 0.02537	4.08457 ± 0.02203
Reduced Chi-Sqr	0.0072	0.11764	0.09397	0.07095	0.15121
R-Square (COD)	0.99684	0.99451	0.99652	0.99671	0.99719
Adj. R-Square	0.99683	0.9945	0.99651	0.9967	0.99719

Figure S9. Comparison of OCP level at different temperatures and $[Na^+]$ levels.

A

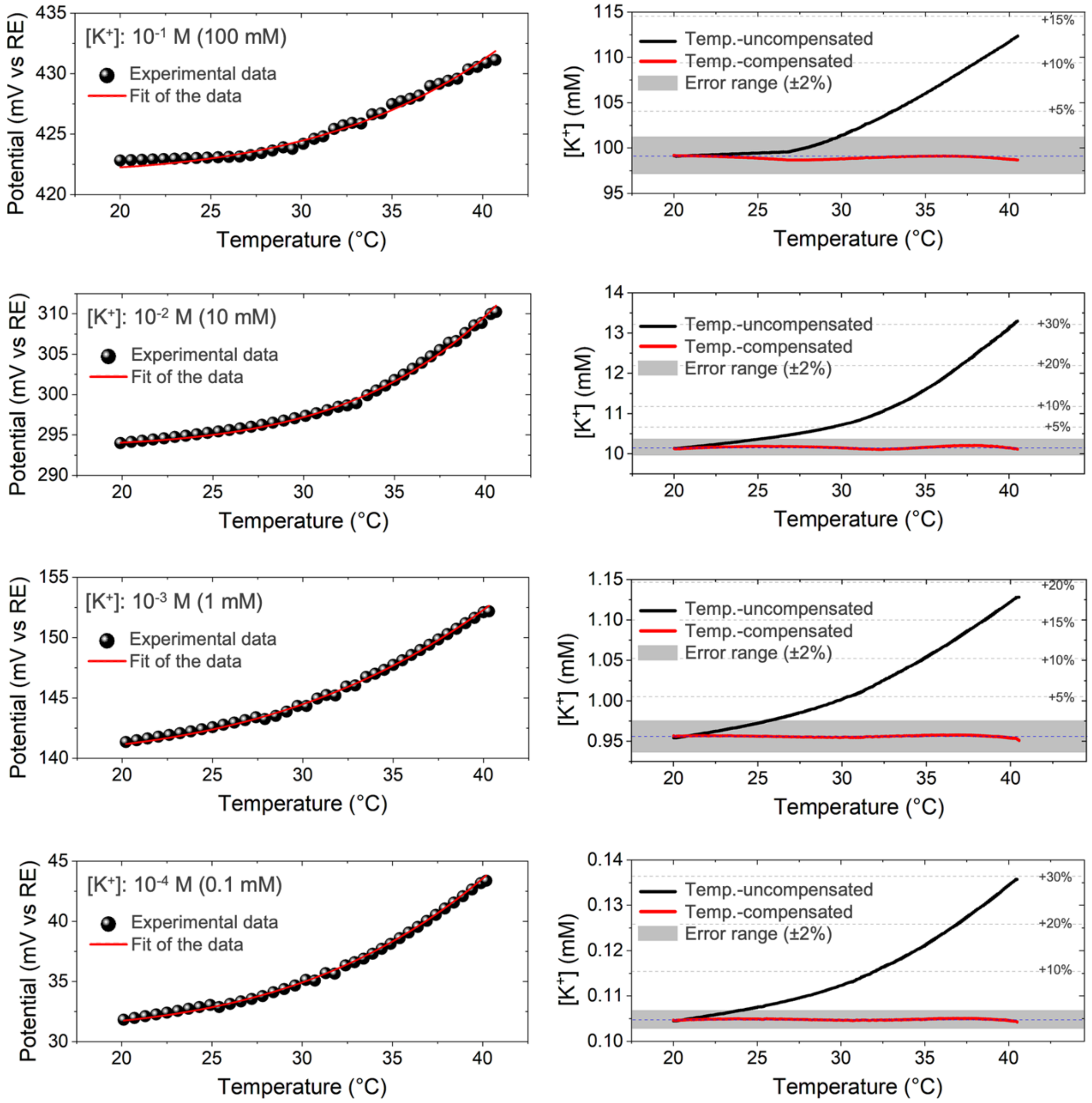

B

Equation	$y = a + bx^c$			
$[K^+]$ (M)	10^{-4}	10^{-3}	10^{-2}	10^{-1}
a	-0.86531 ± 0.02078	-1.23272 ± 0.02602	-0.36938 ± 0.0198	-1.01773 ± 0.03355
b	4.59437E-6 ± 3.08732E-7	2.32612E-5 ± 1.652E-6	7.0655E-8 ± 5.29149E-9	8.73502E-7 ± 1.43455E-7
c	4.01871 ± 0.01801	3.57015 ± 0.01892	5.2167 ± 0.02021	4.38716 ± 0.04407
Reduced Chi-Sqr	0.02339	0.02469	0.04903	0.08621
R-Square (COD)	0.99811	0.99778	0.99798	0.98888
Adj. R-Square	0.99811	0.99778	0.99797	0.98886

Figure S10. Comparison of OCP level at different temperatures and $[K^+]$ levels.

A

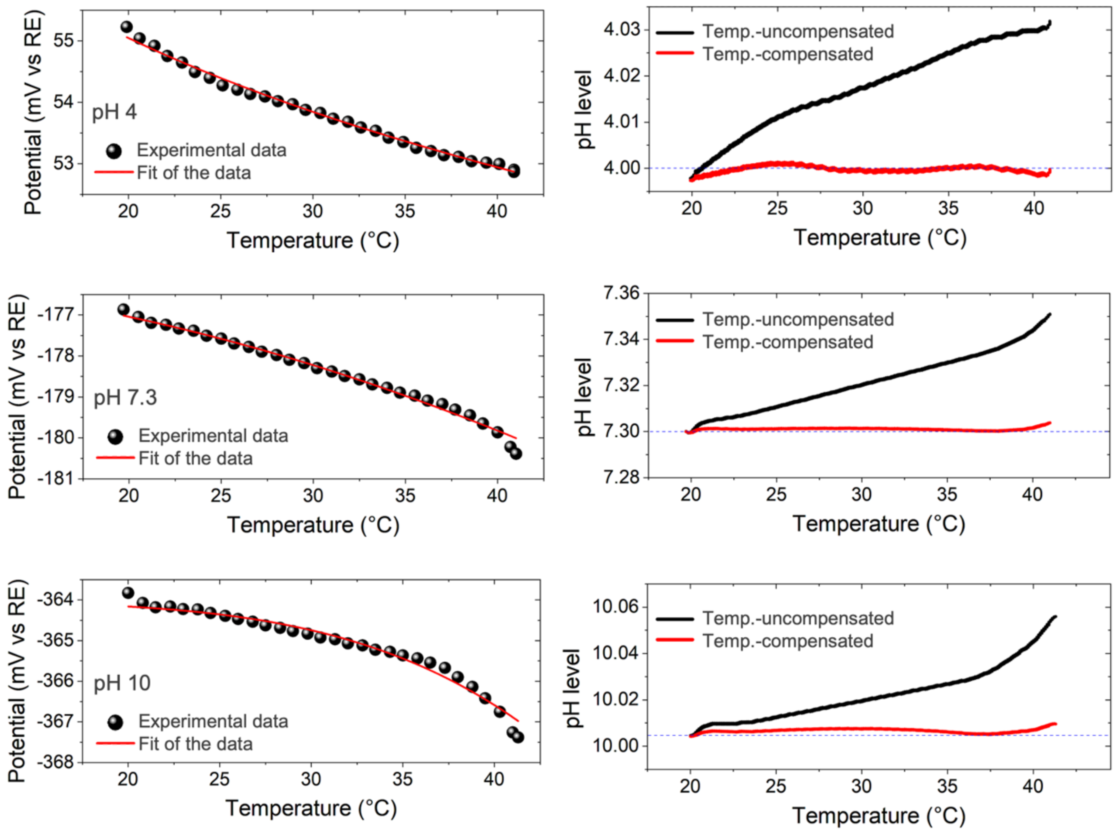

B

Equation	y = a + bx^c		
pH	4	7.3	10
a	17.11377 ± 3.43657	0.84253 ± 0.03947	-0.2239 ± 0.01499
b	-10.53103 ± 3.06524	-0.00345 ± 4.65723E-4	-1.55043E-7 ± 4.26097E-8
c	0.1656 ± 0.03092	1.89832 ± 0.03406	4.50279 ± 0.07364
Reduced Chi-Sqr	0.00276	0.00584	0.01519
R-Square (COD)	0.99307	0.99232	0.97648
Adj. R-Square	0.99305	0.9923	0.97642

Figure S11. Comparison of OCP level at different temperatures and pH levels.

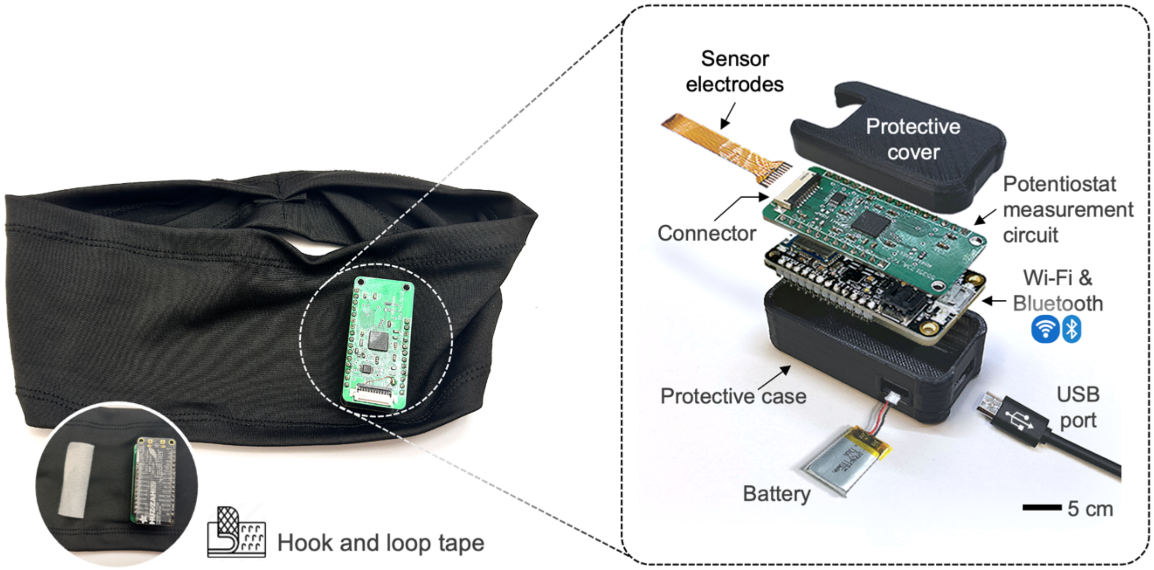

Figure S12. Wireless circuitry system.

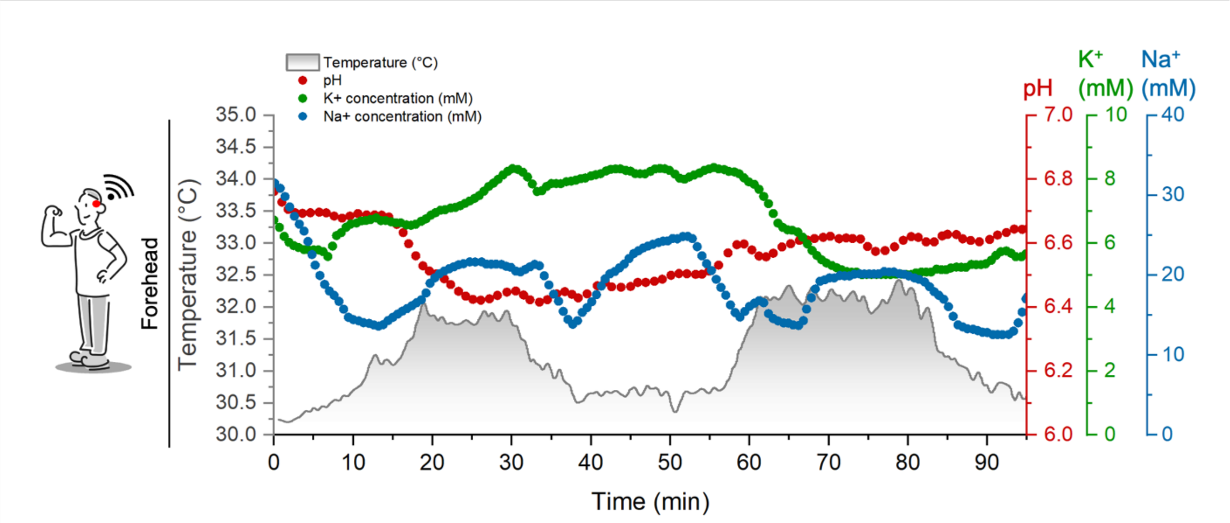

Figure S13. On-body monitoring of forehead sweat electrolyte levels and skin temperature during normal activities. Real-time measurements of forehead skin temperature (gray), pH (red), Na^+ concentration (blue), and K^+ concentration (green) recorded over a 90-minute session involving physical activity.

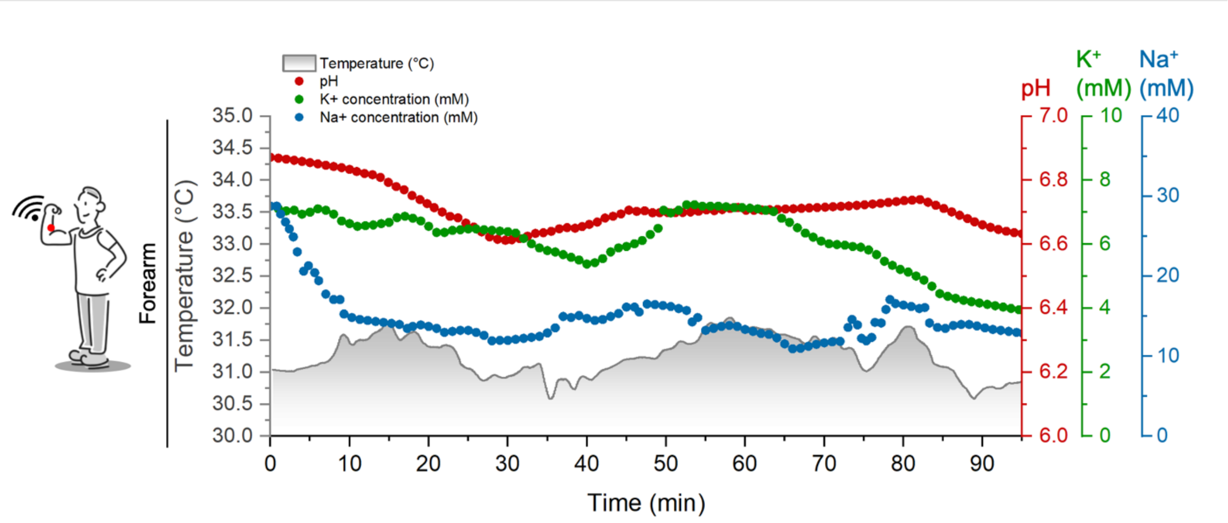

Figure S14. On-body monitoring of forearm sweat electrolyte levels and skin temperature during normal activities. Real-time measurements of forehead skin temperature (gray), pH (red), Na^+ concentration (blue), and K^+ concentration (green) recorded over a 90-minute session involving physical activity.

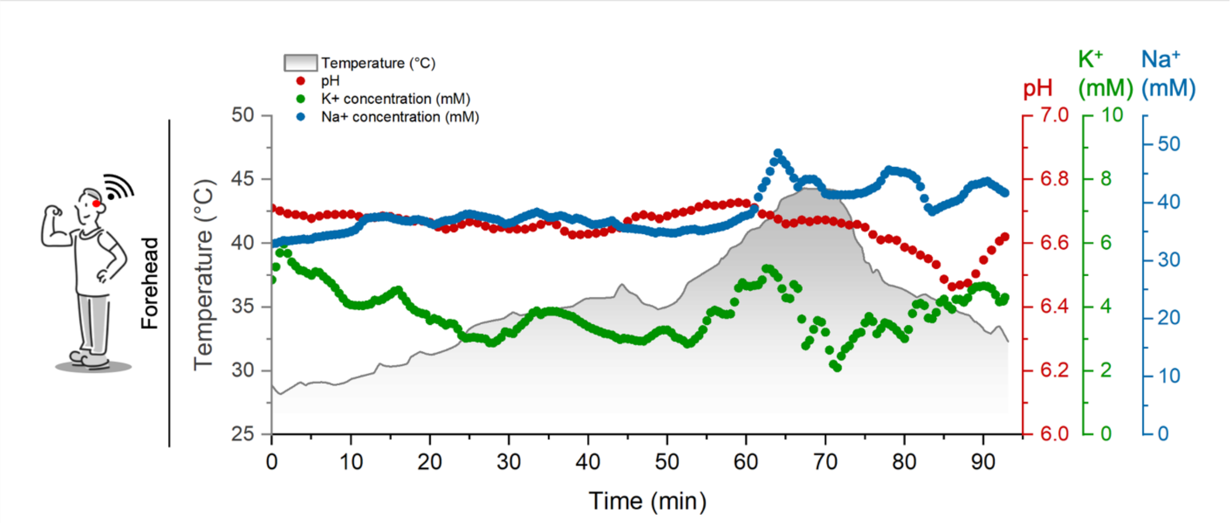

Figure S15. On-body monitoring of sweat electrolyte levels and skin temperature during exercise under harsh temperature variation. Real-time measurements of forehead skin temperature (gray), pH (red), Na^+ concentration (blue), and K^+ concentration (green) recorded over a 90-minute session involving physical activity.

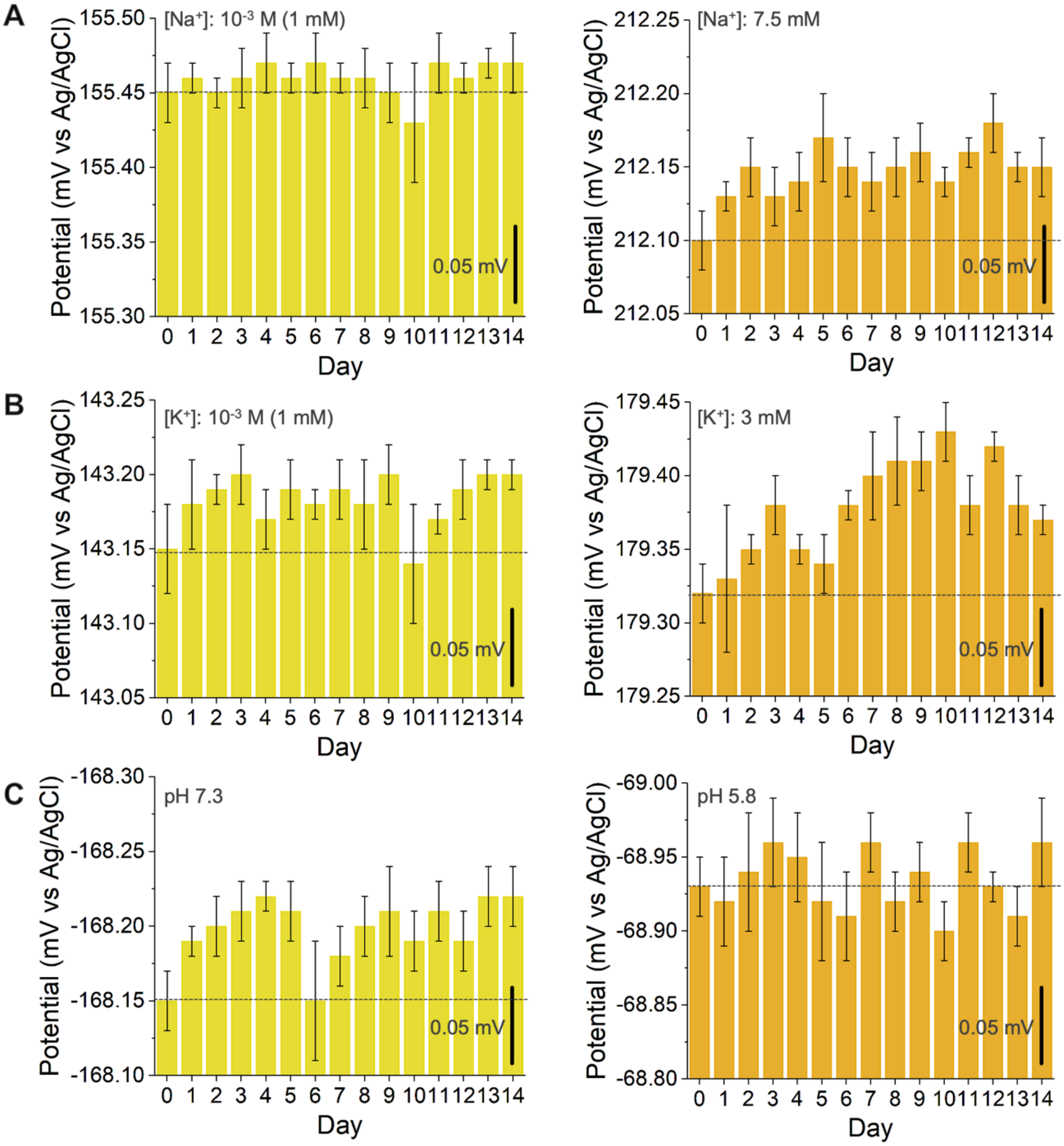

Figure S16. Long-term stability assessment of potentiometric sweat sensors over 14 days in PBS (left column) and artificial sweat (right column). A Na^+ sensor; B K^+ sensor; C pH sensor. All sensors were tested in well-defined calibration solutions (1 mM Na^+, 1 mM K^+, and pH 7.3) under both PBS and artificial sweat conditions.

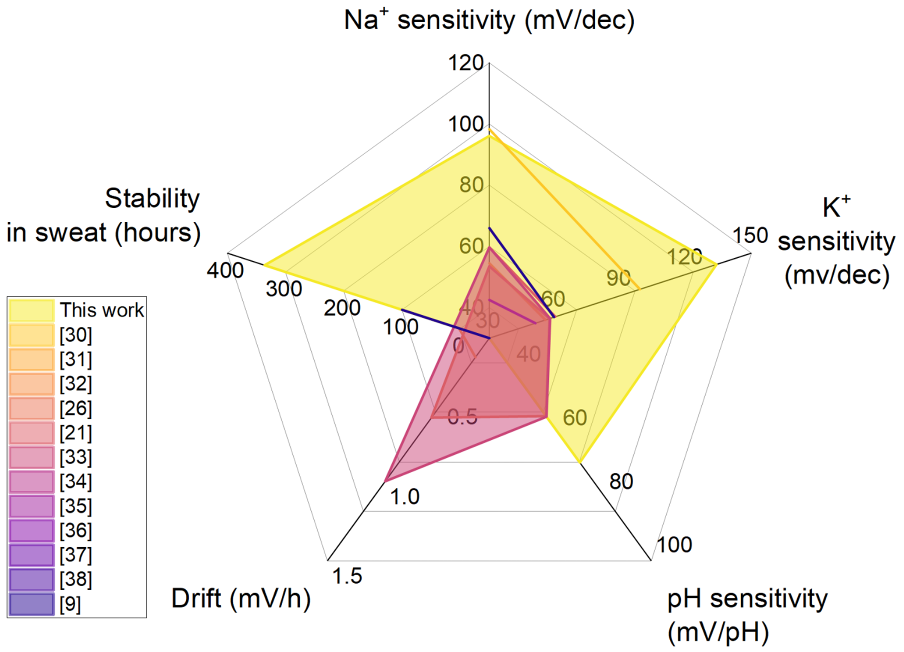

Figure S17. Comparative performance analysis of sweat electrolyte sensors. Radar plot comparing key sensor metrics—Na^+ sensitivity, K^+ sensitivity, pH sensitivity, stability in sweat, and potential drift (mV/h)—for various previously reported sensors and this work. The proposed temperature-compensated sensor (yellow region) demonstrates enhanced stability, superior sensitivity, and lower potential drift compared to conventional sensors. Notably, the exceptional stability in sweat (>300 hours) and minimal drift (<0.15 mV/h) underscore its reliability for long-term wearable applications.
